# Proof of Concept of a Dynamic Energy Prescription Protocol Integrating Wearable Activity Data in 19 Adult Dogs: A Prospective Longitudinal Study

**DOI:** 10.3390/vetsci13050499

**Published:** 2026-05-20

**Authors:** Carina Sacoor, Carolina Domingues, Sara Leitão, Ricardo Cabeças, Felisbina L. Queiroga

**Affiliations:** 1 Vasco da Gama Research Center (CIVG), Vasco da Gama University School (EUVG), 3020-210 Coimbra, Portugal; ricardo.cabecas@euvg.pt; 2 Department of Veterinary Sciences, School of Agrarian and Veterinary Sciences, University of Trás-os-Montes and Alto Douro (UTAD), 5000-801 Vila Real, Portugal; 3 Maven Pet Inc., Wilmington, DE 19801, USA; carolina@maven.pet (C.D.);; 4 Animal and Veterinary Research Center (CECAV), University of Trás-os-Montes and Alto Douro (UTAD), 5001-801 Vila Real, Portugal; 5 Associate Laboratory for Animal and Veterinary Sciences (AL4AnimalS), 1300-477 Lisboa, Portugal

**Keywords:** canine nutrition, maintenance energy requirement, body condition score, wearable sensors, personalized feeding, activity monitoring, dynamic energy prescription

## Abstract

Standard predictive equations for maintenance energy requirements often do not account for individual variability in dogs, potentially leading to nutritional inaccuracies. This prospective proofofconcept study evaluated a dynamic energy prescription protocol integrating wearable activity data in adult shelter dogs. Twenty-six dogs were enrolled, of which 19 had valid longitudinal data for analysis. The 10-week protocol comprised a fixed energy prescription phase (R1), based on perceived activity level, followed by a dynamic phase (R2), in which energy allowance was recalculated weekly incorporating accelerometer-derived activity data and other individual parameters. During R2, BCS remained unchanged in 12/19 dogs and varied one unit in 7/19. Of these, two dogs with above-ideal baseline BCS decreased, and one dog with below-ideal BCS increased. Body weight (mean ± SD: 24.2 ± 7.0 vs. 23.6 ± 6.7 kg; *p* < 0.001) and body fat percentage (19.9 ± 5.4 vs. 18.3 ± 4.6%; *p* = 0.010) decreased, while muscle condition score remained stable. Feeding adherence improved significantly from 8/19 during R1 to 18/19 during R2 (*p* = 0.004). These findings support the feasibility of integrating activity monitoring into a dynamic energy prescription and its potential to improve alignment between prescribed and consumed energy. Longer-term studies are warranted to confirm these observations.

## Simple Summary

Feeding dogs with the right amount of energy is important for their health, but standard feeding guidelines may not suit every individual. This study tested a new approach in 19 shelter dogs, where the amount of food each dog received was adjusted weekly based on data from a wearable sensor that tracked their activity levels, combined with other individual characteristics. Over 10 weeks, most dogs maintained a stable body condition, while a minority varied by one unit, both toward and away from the ideal range. Compared to a standard fixed feeding plan, this dynamic approach resulted in dogs eating more consistently close to their prescribed amount. These findings suggest that using wearable technology to personalize feeding plans could help improve nutritional management in dogs.

## 1. Introduction

Nutritional assessment is recognized as a fundamental component of routine veterinary care for dogs [1]. Appropriate energy prescription is essential in canine nutrition [2], with implications for long-term health and longevity [3], including obesity-related metabolic disturbances, orthopedic disease, and reduced quality of life [4,5]. Traditional recommendations are mainly based on population-level estimates of daily energy requirements (DER), adjusted for factors such as body weight, age, reproductive status, and perceived activity level [6,7]. However, the variability in metabolizable energy requirements observed in clinical settings suggests relevant limitations in relying exclusively on standardized recommendations, and can vary with neutering status and Body Condition Score (BCS) [8]; also, activity levels and husbandry are factors that reflect substantial inter-individual variability in estimated energy requirements [9]. Breed, reproductive status, bedding location, and temperament are often underrepresented in conventional models, and have been identified as relevant determinants that improve the accuracy of maintenance energy requirements (MER) estimation in dogs [10]. In addition, dogs with higher BCS have markedly lower MER than those predicted by reference tables, reinforcing the importance of incorporating body condition into energy prescription [2]. These limitations are further compounded by wide variability in commercial feeding guides, which utilize heterogeneous predictive approaches and have been characterized as conservative estimates [11].

The development of wearable activity sensors for dogs has been enabling the objective, continuous quantification of specific activity and locomotion states, such as sleeping, inactivity, walking, trotting, galloping and others. Performance was expressed using different metrics depending on the target behavior and study design, including agreement with direct observation ranging from 75% to 99% [12] and classification accuracies between 80% and 99% [13,14,15], in line with an earlier study based on accelerometer data that reported approximately 70% overall classification accuracy across 17 activities [16].

Integrating these activity data from wearable sensors into energy prediction models has been proposed to improve estimates of energy expenditure, compared to conventional approaches relying on generic activity factors, thereby reducing the discrepancy between predicted needs and the actual intake required [17]. By continuously monitoring activity, these technologies also enable the detection of temporal fluctuations in activity patterns over time [18].

Despite the growing interest in wearable devices for dogs, dynamic energy prescription systems based on sensor data remain understudied and their predictive accuracy investigated in some studies has not been demonstrated [17,19]. In human research, accelerometer activity data have been evaluated for estimating energy expenditure; however, agreement with criterion methods is often limited, and heterogeneity in performance has been reported across devices, sensor placement, activity types, and analytical models [20,21,22,23,24].

We propose that objective activity data, combined with other individual variables, can be incorporated into a dynamic prescription algorithm to more closely match energy intake with each dog’s actual requirements. Therefore, the present proof-of-concept study aimed to evaluate the feasibility and preliminary outcomes of a dynamic energy prescription regimen in healthy adult dogs, following a fixed-prescription phase. Data from the fixed phase are presented as contextual reference, while the primary analysis focuses on the responses observed during the dynamic phase. This study seeks to contribute to the understanding of how such systems perform in practice and to identify elements relevant for further refinement and clinical validation.

## 2. Materials and Methods

### 2.1. Study Design

The study was conducted between 29 September and 15 December 2025 in healthy dogs housed at the Official Animal Collection Centre (CROA), Porto, Portugal, over a 10-week period. The protocol comprised two sequential feeding regimens: an initial fixed energy prescription phase, Regime 1 (R1), followed by a dynamic energy prescription phase, Regime 2 (R2). Although data from both regimens are presented for contextual reference, the analysis focuses on longitudinal responses during R2. The study was approved by the Ethics Committee of the University of Trás-os-Montes e Alto Douro (Doc46-CE-UTAD-2025).

### 2.2. Population

Twenty-six adult dogs housed at CROA were enrolled. Dogs were maintained in individual kennels and had daily access to indoor and outdoor yards for 30 min. Inclusion criteria comprised medium-sized, neutered adult dogs (>1 year), clinically stable, based on veterinary examination, without diagnosed conditions, and not receiving therapeutic diets. Baseline BCS and habitual diet were not predefined as inclusion criteria. Exclusion criteria included dogs presenting impaired locomotion, associated with musculoskeletal or other medical conditions, as determined by veterinary evaluation.

### 2.3. Study Phases and Feeding Regimens

The study comprised three consecutive phases over a 10-week period.

During the baseline phase (weeks 1–2), dogs were fed according to a fixed energy prescription based on age-specific current recommendations for healthy adult dogs [4]. This phase served to collect reference data and document baseline dog daily routines.

In R1 (weeks 3–6), a fixed energy prescription was implemented, with DER determined according to perceived activity level, in line with current recommendations [4]. Daily energy intake (kcal/day) was determined at the beginning of this phase and maintained constant throughout the four-week period.

In R2 (weeks 7–10), a dynamic energy prescription was implemented using a continuous-variable algorithm developed by Maven Pet^®^ (Wilmington, DE, USA; http://maven.pet, accessed on 1 May 2026). The algorithm first established a baseline energy requirement by applying standard metabolic scaling to body weight, which was subsequently adjusted according to individual variables including BCS, breed size, life stage, reproductive status, and health status. Sensor-derived activity data, expressed as weekly mean time spent in the ‘active’ and ‘excited’ states, were incorporated as a modulating factor, with the two activity intensities differentially weighted so that higher-intensity activity exerted a greater influence on the final energy estimate. The resulting individualized daily energy requirement (kcal/day) determined the amount of food offered to each dog and was recalculated weekly throughout R2 to reflect changes in activity patterns.

### 2.4. Food Intake Monitoring

Dogs were fed commercially available dry kibble once daily. The diets used were those already provided by the facility prior to the study, selected by the municipal veterinary team in accordance with institutional procurement criteria. No dietary changes were introduced for the purposes of this study. Three formulations were used depending on individual requirements as determined by the facility’s veterinary team: Ownat Classic Complet (adult maintenance; crude protein 25%, fat 12%, ME 3650 kcal/kg), Ownat Classic Junior (growth; crude protein 28%, fat 14%, ME 3810 kcal/kg), and Ownat Classic Mini Adult (small-breed adult; crude protein 26%, fat 13%, ME 3700 kcal/kg) (Ownat, Cotécnica, Bellpuig, Spain). The dogs did not have ad libitum access to food. Each dog received an individualized daily portion corresponding to the prescribed energy allowance for the respective regimen (R1 or R2). Food portions were weighed using a digital scale (precision ±1 g) and provided at 8 AM. After 24 h, bowls were removed and the remaining food was weighed and recorded for each dog. Daily food intake was calculated as the difference between the amount offered and the amount remaining. All weighings were performed by the same veterinarian. Intake was recorded daily, and weekly mean intake for each dog was calculated for analysis. Feeding adherence was also assessed and defined as a weekly mean intake of at least 90% of the prescribed food amount. Adherence was considered consistent when this criterion was met in all weeks of the respective regimen.

### 2.5. Activity Monitoring

For R1, perceived activity level was independently classified by two veterinarians and one animal caretaker, who were directly involved in the dogs’ daily management and familiar with their routines. The final activity category used to determine the fixed DER corresponded to the modal classification, the most frequently assigned category.

The activity of the dogs was continuously monitored throughout the study using the Maven Pet^®^ AI System. The system comprises a collar-mounted sensor equipped with a three-axis accelerometer and three-axis gyroscope, enabling continuous collection of movement data that was subsequently processed to quantify activity levels. The sensor was positioned on the ventral aspect of the dog’s collar and was worn continuously throughout the study period, with brief removal only for routine battery charging. Movement data were sampled at 104 Hz and processed at the sensor level every 15 s, then synchronized with a cloud-based platform every 15 min.

Activity data were automatically classified by the device’s algorithm into four predefined activity states based on accelerometry- and gyroscopy-derived motion intensity: resting (inactivity, without distinction between ‘awake and resting’ and ‘sleeping’), quiet (minimal activity without locomotion), active (sustained low intensity activity), and excited (high intensity activity), as previously described [18]. For analysis, time spent in the ‘active’ and ‘excited’ states was calculated as weekly mean values and used as input parameters in the dynamic energy prescription during R2.

### 2.6. Body Parameters Evaluation

Body parameters were assessed weekly throughout the study. Body weight was measured using a calibrated veterinary scale (Lubb, Ponte de Lima, Portugal). BCS was independently assessed by two veterinarians using a standardized nine-point scale [1,25]. Muscle condition score (MCS) was also assessed by the same evaluators [26]. Body fat percentage (BF%) was estimated using a validated morphometric equation incorporating morphometric measurements such as pelvic circumference and hock-to-stifle length, as described by Witzel et al. [27], providing a quantitative measure to complement the clinical BCS assessment.

### 2.7. Statistical Analysis

All analyses were conducted in the statistical program R, version 4.3.3 [28]. Descriptive analysis and data visualization were performed for body parameters and activity across study phases. Activity data were aggregated as weekly mean values per dog. The BCS score was categorized as decrease, maintenance, or increase based on the difference between the beginning and end of each feeding regimen, and results were summarized as absolute frequencies and percentages. To compare the distribution of dogs across BCS change categories between regimens, Pearson’s chi-square test was applied. Given small cell counts in some categories, *p* values were confirmed using Fisher’s exact test. For comparison between regimens, mean weekly time spent in the active and excited states was calculated for each dog during R1 and R2 and compared using Wilcoxon signed-rank tests. Wilcoxon signed-rank tests were used for other paired comparisons where appropriate. Data are presented as mean ± standard deviation (SD). The level of statistical significance was set at α = 0.05.

## 3. Results

### 3.1. Study Population

Nineteen dogs were included in the final analysis following the exclusion of seven animals. Reasons for exclusion included inability to accurately quantify food intake (*n* = 3), adoption during the study period (*n* = 2) preventing consistent monitoring; chronic diarrhea with potential to confound outcomes (*n* = 1), and death from causes unrelated to the study (*n* = 1). Most dogs were mixed-breed (*n* = 15), with Fila Brasileiro (*n* = 1) and Pit Bull (*n* = 1) breeds also represented (Table 1).

### 3.2. Body Parameters

**Figure 1:**
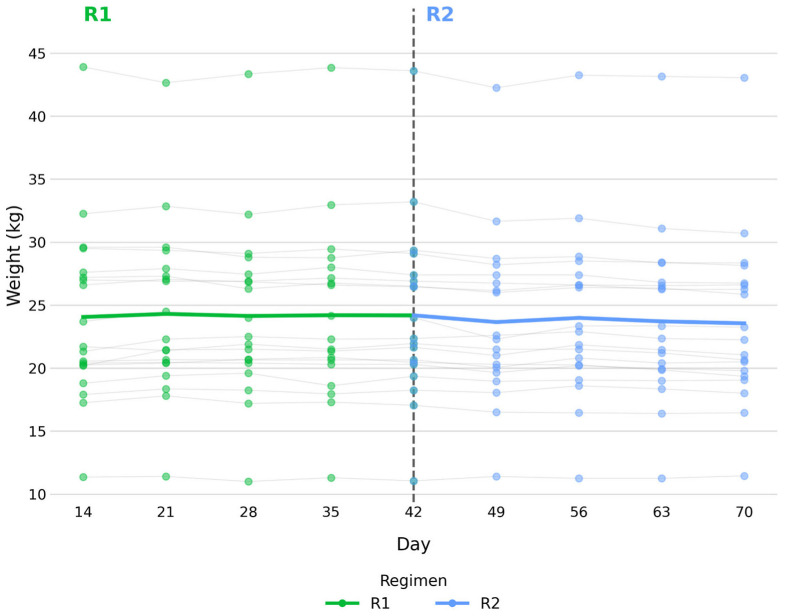
Individual longitudinal body weight trajectories for each dog across the study period.

Figure 1 shows individual longitudinal body weight trajectories across the study period, illustrating the variability between dogs and variations within each dog. During R1, mean body weight was 24.1 ± 7.1 kg at baseline and 24.2 ± 7.0 kg at endpoint (Wilcoxon signed-rank test, *p* = 0.573). During R2, mean body weight was 24.2 ± 7.0 kg at baseline and 23.6 ± 6.7 kg at endpoint (Wilcoxon signed-rank test, *p* < 0.001).

**Figure 2:**
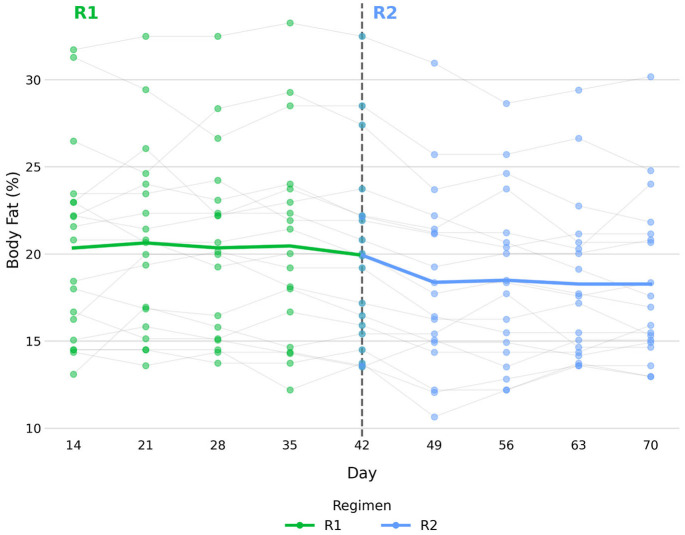
Longitudinal BF% trajectories for each dog across the study period.

Figure 2 shows individual longitudinal trajectories of BF% across the study period. During R1, mean BF% was 20.3 ± 5.5% at baseline and 19.9 ± 5.4% at endpoint (Wilcoxon signed-rank test, *p* = 0.310). During R2, mean BF% was 19.9 ± 5.4% at baseline and 18.3 ± 4.6% at endpoint (*p* = 0.010).

Table 2 summarizes BCS at the beginning and end of each regimen and the corresponding variation per dog. During R1, BCS decreased in 3/19 dogs, remained unchanged in 15/19, and increased in 1/19. During R2, BCS decreased in 6/19 dogs, remained unchanged in 12/19, and increased in 1/19. No changes greater than one BCS unit were observed. In Table 3, the distribution of dogs across the decrease, unchanged, and increase categories did not differ between regimens (*p* = 0.513). Given the low count in some cells, the absence of difference was confirmed by Fisher’s exact test (*p* = 0.714). The MCS remained unchanged in all dogs during both regimens.

**Figure 3:**
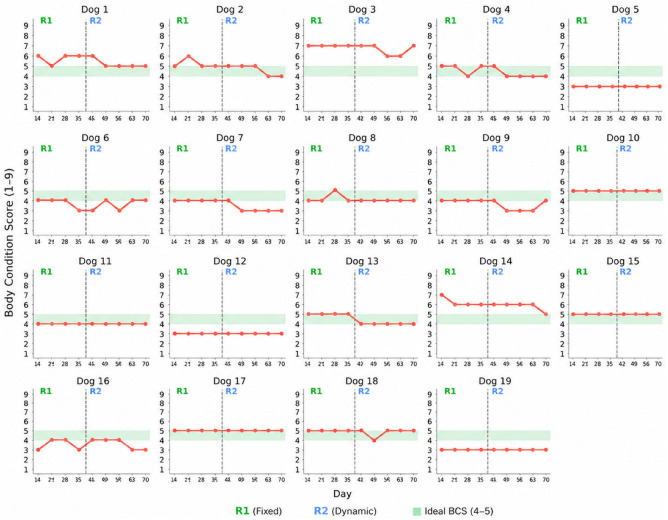
Individual BCS trajectories across the study period.

Figure 3 shows individual BCS trajectories across the study period, illustrating overall stability, short term fluctuations, and each dog’s position relative to the ideal BCS range (4–5/9) [29].

### 3.3. Activity

**Figure 4:**
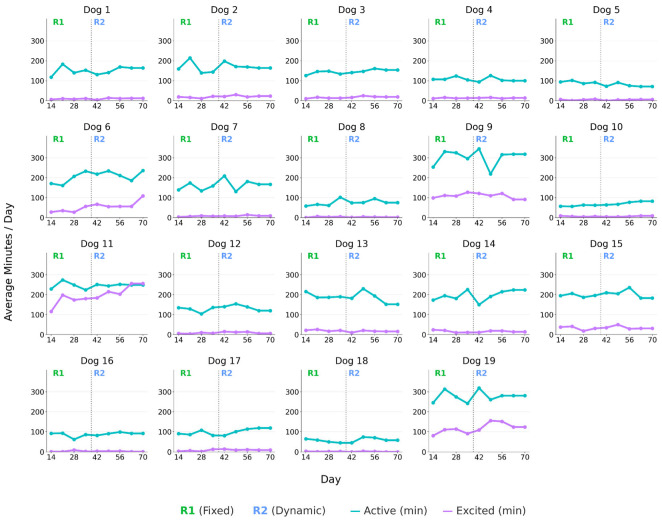
Individual weekly mean minutes per day spent in the active and excited states across the study period.

Figure 4 shows individual weekly mean activity trajectories for the active and excited states across the study period. Weekly mean time spent in the active state was 153.1 ± 73.5 min/day during R1 and 158.8 ± 72.1 min/day during R2 (Wilcoxon signed-rank test: *p* = 0.020). Time spent in the excited state was 30.9 ± 46.2 min/day during R1 and 37.4 ± 57.8 min/day during R2 (*p* = 0.036). Activity patterns also varied between dogs and across weeks within individual dogs, as illustrated in Figure 4.

### 3.4. Feeding Plan Adherence and Energy Intake

**Figure 5:**
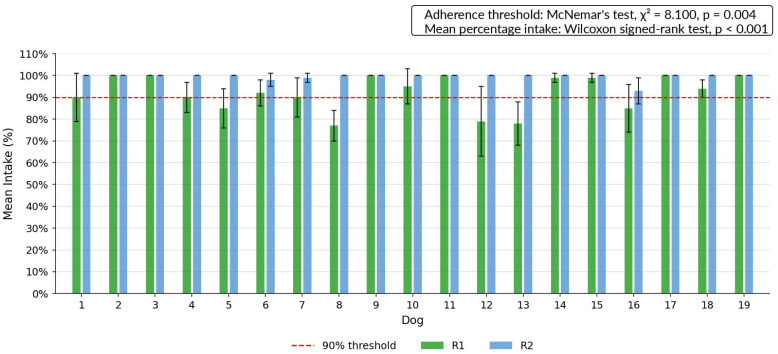
Mean weekly percentage of kilocalorie intake relative to the prescribed amount for each dog during R1 and R2. Error bars represent standard deviation. The dashed red line indicates the 90% adherence threshold. The proportion of dogs consistently meeting the adherence threshold was significantly higher during R2 (McNemar’s test: χ^2^ = 8.100, *p* = 0.004) Mean percentage intake also differed significantly between regimens (Wilcoxon signed-rank test: *p* < 0.001).

**Figure 6:**
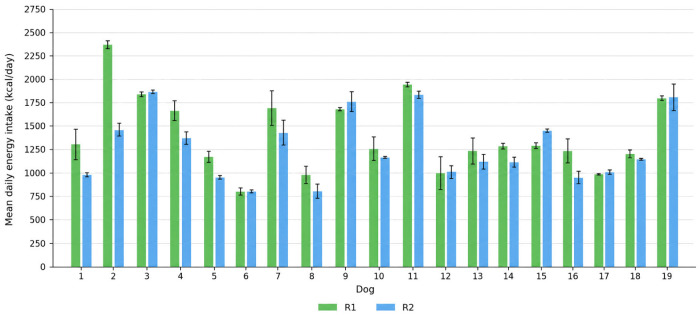
Mean daily metabolizable energy intake (kcal/day) per dog during R1 and R2. Each bar represents the mean of the four weekly intake values within the corresponding regimen. Error bars represent the standard deviation across the four weeks.

Across the study period, consistent feeding plan adherence, defined as weekly mean intake ≥90% of the prescribed amount in all weeks, was observed in 8/19 dogs during R1 and in 18/19 dogs during R2. Mean percentage intake relative to the prescribed amount was 92% (SD = 8; range: 78–100%) during R1 and 99% (SD = 2; range: 93–100%) during R2. The proportion of dogs meeting the adherence threshold was significantly higher during R2 (McNemar’s test: χ^2^ = 8.100, *p* = 0.004), and the distribution of mean percentage intake also differed significantly between regimens (Wilcoxon signed-rank test: *p* < 0.001). Individual dietary adherence by animal and regimen is illustrated in Figure 5. Mean daily metabolizable energy intake per dog during each regimen is illustrated in Figure 6.

## 4. Discussion

This prospective longitudinal study evaluated the implementation of a dynamic energy prescription protocol integrating objective activity monitoring in a real-world shelter setting. The findings provide preliminary insights into the feasibility and short-term responses associated with this approach.

The study population included a broad age range and was predominantly mixed-breed (Table 1), reflecting the heterogeneity typical of a municipal shelter setting and enhancing the real-world relevance of these observations.

BCS is a central parameter for assessing nutritional status in dogs [1]. In adult dogs, body weight is an objective and reproducible parameter for longitudinal monitoring; however, body weight can vary considerably within a breed and does not distinguish between changes in body composition [30]. Therefore, in this study, body weight was interpreted together with BCS, MCS, and estimated BF% to provide a broader assessment of the dogs’ response to the feeding regimens.

During R2, mean body weight remained decreased at the group level (*p* < 0.001), while individual variability in baseline values and longitudinal trajectories were observed across dogs (Figure 1). Overall, the transition to R2 was not accompanied by clinically relevant changes in body condition. No detectable change in BCS was observed in 12/19 dogs (Table 2), although subtle variations within the resolution of the 9-point scale cannot be excluded. Occasional transient and reversible fluctuations were observed in the remaining dogs (Figure 3).

Changes in body condition were interpreted in relation to baseline BCS, with the primary changes observed representing favourable shifts in dogs starting above or below the ideal range. Given the small number of dogs showing BCS changes and the limited number of dogs starting outside the ideal range, these observations should be considered descriptive and exploratory rather than evidence of a regimen-specific effect. During R2, two dogs with an elevated baseline BCS showed a one-unit decrease, resulting in scores closer to the ideal range. This observation aligns with recent findings by Pedrinelli et al., 2021, who demonstrated a negative association between BCS and MER in a retrospective study, with a 9.8 kcal/kg^0.75 decrease in energy requirements for each one-unit increase on the 9-point scale, regardless of diagnosis [2]. A similar pattern was evident in clinical settings, where overweight and obese dogs had substantially lower mean energy factors than dogs with an ideal BCS [8]. In this context, the caloric adjustments implemented during R2 appear compatible with previously described associations between BCS and MER, although the limited number of dogs precludes definitive conclusions regarding the effect of R2 on body condition. The increase in one-unit BCS observed in one dog with below-ideal baseline BCS may reflect a clinically appropriate shift toward the ideal range, reinforcing the need to interpret individual trajectories in relation to baseline status, while acknowledging that single-animal observations cannot be generalized. Two dogs at the upper end of the ideal range remained within it (dogs 2 and 4) and two dogs at the lower end of the ideal range decreased one unit (dogs 7 and 16), although it was not accompanied by clinically detectable muscle loss. These individual-level observations are exploratory in nature and should be confirmed in larger cohorts before drawing firm inferences. MCS remained stable in all dogs throughout the study period, indicating that the changes observed in these four dogs were most likely attributed to reductions in fat mass. The distribution of BCS change categories did not differ significantly between regimens (Table 3), indicating that R2 did not produce a meaningful shift in body condition. Additionally, mean BF% decreased during R2 (*p* = 0.010), generally in the context of unchanged BCS in most dogs (Figure 2 and Figure 3), suggesting a closer alignment between prescribed and actual energy requirements. It should also be acknowledged that BCS is a semi-quantitative clinical assessment and is less sensitive than body weight or morphometric measurements for detecting small short-term changes. Therefore, the one-unit BCS variations observed in this study should be interpreted cautiously, as such changes may fall within the expected variability of clinical scoring. For this reason, BCS changes were interpreted alongside body weight, BF%, and MCS rather than as isolated evidence of body composition change.

Overall, the heterogeneity observed in the response to the dynamic regimen (R2) is consistent with known variability in MER and underscores the importance of individualized energy prescription. An allometric model identified breed, reproductive status, resting location, and temperament as variants for MER, requiring correction factors ranging from 0.85 to 1.09 relative to the base equation, thereby supporting the integration of multiple parameters when calibrating individual energy intake [10]. Beyond these intrinsic factors, previous work has suggested that objectively measured activity has the potential to provide an additional source of variation relevant to individual energy calibration [17]. In this study, sensor-derived weekly activity trajectories varied between dogs and displayed marked fluctuations within individuals over time (Figure 4). Mean time spent in both the active and excited states was higher during R2 than during R1; however, although statistically significant, these differences were modest and should be interpreted cautiously given the short duration of each regimen and the self-controlled before–after design.

These activity patterns are relevant when interpreting differences between R1 and R2, because the two regimens differed in how activity was incorporated into the energy prescription. In R1, energy prescription was based on a fixed perceived activity category assigned to each dog, consistent with current clinical practice. Although misclassification by the assessors cannot be completely excluded, this risk was minimized by independent classification from two veterinarians and one animal caretaker familiar with the dogs’ routines. In addition, even when correctly assigned, a fixed perceived activity category has inherently limited granularity and cannot account for temporal fluctuations in activity compared with continuous sensor-derived data. Therefore, the R1 approach may have provided a less precise representation of actual activity patterns, which may have contributed to differences in body parameters observed between R1 and R2, including body weight changes.

Despite the potential of sensors to quantify activity in dogs, converting movement data into energy expenditure estimates critically depends on the underlying algorithm. Furthermore, the currently available literature on this topic remains limited and inconsistent. In a 28-day evaluation study using a commercial wearable monitor, poor agreement was observed between device-estimated DER and observed DER, with overestimation in 78.3% of dogs and only 8.6% meeting a ≤5% difference criterion [19]. By contrast, a pilot study (indexed journal publication available only as an abstract) described a mean absolute error decreasing from approximately 103 to 86 kcal/day when wearable data activity was incorporated, in comparison with a standard approach using a fixed activity factor [17].

Feeding plan adherence was significantly higher during R2, both in the proportion of dogs consistently meeting the 90% intake threshold and in mean percentage intake (Figure 5). This difference is unlikely to be attributable to weighing error, as food portions and leftovers were systematically weighed in both phases [31]. However, feeding adherence reflects the agreement between prescribed and consumed food, and should not be interpreted as direct evidence of greater accuracy in estimating true energy requirements. Rather, the higher adherence observed during R2 suggests that the dynamic prescription may have produced food allocations that were more consistently consumed by the dogs. Whether this reflects closer alignment with individual energy requirements requires further validation using objective measures of energy expenditure and longer-term body composition outcomes. In addition, given dogs’ propensity to maximize energy intake, voluntary consumption can increase with portion size, potentially promoting overconsumption within a single meal [32]. In this context, the lower intake variability observed during R2, alongside reduced voluntary feed refusal, indicates a more consistent agreement between prescribed and consumed energy across the cohort. The individual mean daily ME intake values further illustrate substantial between-dog variability in absolute kcal intake across regimens (Figure 6). This pattern is particularly relevant in shelter or group-housed settings, where standardized feeding routines are common and individual adjustments are often limited. These observations on feeding adherence merit further evaluation in broader cohorts.

This study has several limitations. The sample size was small and restricted to a shelter population, which may limit generalizability to client-owned dogs. The study duration was relatively short and does not allow conclusions regarding long-term body composition changes. In addition, energy metabolism-related markers, such as glucose and lipid profiles were not collected, energy expenditure was not measured using a gold-standard method, and the proprietary algorithm was not externally validated within this study. Therefore, these findings should be interpreted as preliminary and hypothesis-generating. The observed improvements in feeding adherence and the short-term changes in body weight and estimated BF% may suggest the feasibility of implementing a dynamic prescription framework in this setting, but they should not be interpreted as definitive evidence of improved accuracy in estimating individual energy requirements. Confirmation in larger and longer-term studies, ideally including objective measures of energy expenditure and body composition as well as additional independent BCS assessors, is required before effectiveness or predictive accuracy can be established.

## 5. Conclusions

The patterns observed during R2 reflect a dynamic prescription framework that enables individual adjustment by incorporating important factors such as BCS, size, breed, neuter status, as well as objective activity data measured by the sensor. This approach may contribute to refining energy prescription by incorporating individual variability that is unlikely to be addressed by static recommendations. The significant improvement in feeding plan adherence during R2 indicates more consistent consumption of prescribed amounts; however, whether this corresponds to closer alignment with actual energy requirements requires confirmation in studies using objective measures of energy expenditure. Larger and longer-duration studies are warranted to evaluate the longer-term effects of dynamic versus fixed feeding regimens.

## Figures and Tables

**Table 1:** Summary of study animal characteristics.

Animal	Breed	Sex	Age (y m)
1	Mixed-breed	F	5 y
2	American Staffordshire cross	M	5 y
3	São Miguel Cattle Dog	M	10 y 1 m
4	Pit Bull cross	M	8 y 8 m
5	Mixed-breed	M	7 y
6	Mixed-breed	M	6 y
7	Mixed-breed	M	9 y 4 m
8	Mixed-breed	F	12 y
9	Mixed-breed	M	2 y 9 m
10	Mixed-breed	M	13 y
11	Pitt Bull	F	4 y 3 m
12	Mixed-breed	M	12 y
13	Mixed-breed	M	5 y 10 m
14	Mixed-breed	F	8 y
15	Mixed-breed	F	7 y 11 m
16	Mixed-breed	M	11 y 7 m
17	Mixed-breed	M	14 y 4 m
18	Mixed-breed	M	9 y
19	Mixed-breed	M	3 y 7 m

**Table 2:** Individual BCS at the beginning and end of each feeding regimen.

Animal	Start R1	End R1	ΔR1	Start R2	End R2	ΔR2
1	6	6	0	6	5	−1
2	5	5	0	5	4	−1
3	7	7	0	7	7	0
4	5	5	0	5	4	−1
5	3	3	0	3	3	0
6	4	3	−1	3	4	+1
7	4	4	0	4	3	−1
8	4	4	0	4	4	0
9	4	4	0	4	4	0
10	5	5	0	5	5	0
11	4	4	0	4	4	0
12	3	3	0	3	3	0
13	5	4	−1	4	4	0
14	7	6	−1	6	5	−1
15	5	5	0	5	5	0
16	3	4	+1	4	3	−1
17	5	5	0	5	5	0
18	5	5	0	5	5	0
19	3	3	0	3	3	0

**Table 3:** Distribution of dogs across BCS change categories by regimen.

Category	R1 *n*	R2 *n* (%)
Decrease	3	6
No change	15	12
Increase	1	1
Total	19	19

Fisher’s exact test: *p* = 0.714.

## Data Availability

The original contributions presented in this study are included in the article. Further inquiries can be directed to the corresponding authors.

## Abbreviations

The following abbreviations are used in this manuscript:

BCS	Body Condition Score
BF%	Body fat percentage
DER	Daily energy requirement
MER	Maintenance energy requirement
MCS	Muscle condition score
R1	Regime 1 (fixed energy prescription phase)
R2	Regime 2 (dynamic energy prescription phase)
CROA	Official Animal Collection Centre
